# Retraction: Up-regulated microRNA-500a enhances hepatocarcinoma metastasis by repressing PTEN expression

**DOI:** 10.1042/BSR-2017-0837_RET

**Published:** 2023-04-21

**Keywords:** AKT, Hepatocarcinoma, Metastasis, MiR-500a

This Retraction follows an Expression of Concern relating to this article previously published by Portland Press. This article is being retracted from *Bioscience Reports*, at the request of the Editor-in-Chief and the Editorial Board, following receipt of a notification from a reader alerting the Editorial Board to an image that seems to also appear in other papers by unrelated authors.

Specifically, the Figure 3C Inhibitor NC panel seems to appear as the Figure 2D Si-Tcf3#3 image from He et al. 2019 (doi: 10.1042/bsr20180369) and as the 143B/sh-VAV3+inhibitor panel of Figure 6E from Xiao et al. 2020 (doi: 10.18632/aging.103762).

The authors have been contacted with regards to the retraction and have not responded to the Journal's queries or the concerns raised. Given the extent of the issues raised, the Editorial Board stand by the decision to retract the article.

